# Relationships between the Decomposition Behaviour of Renewable Fibres and Their Reinforcing Effect in Composites Processed at High Temperatures

**DOI:** 10.3390/polym13244448

**Published:** 2021-12-18

**Authors:** Janez Slapnik, Thomas Lucyshyn, Gerald Pinter

**Affiliations:** 1 Faculty of Polymer Technology, Ozare 19, 2380 Slovenj Gradec, Slovenia; janez.slapnik@ftpo.eu; 2 Montanuniversität Leoben, Otto Glöckel-Straße 2/II, 8700 Leoben, Austria; gerald.pinter@unileoben.ac.at

**Keywords:** thermoplastic composites, renewable fibres, natural fibres, man-made cellulose fibres, injection moulding, thermogravimetric analysis, mechanical properties

## Abstract

Engineering polymers reinforced with renewable fibres (RF) are an attractive class of materials, due to their excellent mechanical performance and low environmental impact. However, the successful preparation of such composites has proven to be challenging due to the low thermal stability of RF. The aim of the present study was to investigate how different RF behaves under increased processing temperatures and correlate the thermal properties of the fibres to the mechanical properties of composites. For this purpose, hemp, flax and Lyocell fibres were compounded into polypropylene (PP) using a co-rotating twin screw extruder and test specimens were injection moulded at temperatures ranging from 180 °C to 260 °C, with 20 K steps. The decomposition behaviour of fibres was characterised using non-isothermal and isothermal simultaneous thermogravimetric analysis/differential scanning calorimetry (TGA/DSC). The prepared composites were investigated using optical microscopy (OM), colorimetry, tensile test, Charpy impact test, dynamic mechanical analysis (DMA) and melt flow rate (MFR). Composites exhibited a decrease in mechanical performance at processing temperatures above 200 °C, with a steep decrease observed at 240 °C. Lyocell fibres exhibited the best reinforcement effect, especially at elevated processing temperatures, followed by flax and hemp fibres. It was found that the retention of the fibre reinforcement effect at elevated temperatures can be well predicted using isothermal TGA measurements.

## 1. Introduction

In the last decade, polymers reinforced with renewable fibres (RF) received much attention both from industry and academia, as they can provide more sustainable alternatives to conventional composites. Lignocellulosic (LCF) and cellulosic fibres (CF) are especially attractive RF due to their large abundance, high specific mechanical properties, biodegradability, low carbon footprint and low abrasion of processing equipment. LCF and CF are often compounded into thermoplastic polymers by extrusion and further processed by injection moulding (IM). However, despite a whole range of attractive properties, the production of such composites presents many challenges, namely, poor interfacial matrix-fibre interactions, high moisture uptake and low thermal stability of fibres. It is generally believed that LCF and CF start to decompose at around 200 °C. Therefore, most of the past research has focused on the preparation of composites based on various thermoplastic polymers that melt below 200 °C, such as polylactic acid (PLA), polyhydroxyalkanoates (PHA), thermoplastic starch (TPS), polyethylene (PE) and polypropylene (PP) [1,2,3,4]. Although these composites have good mechanical and thermal performance, they cannot compete with composites based on engineering polymers, such as PA 6 [5,6], PA 6.10 [6], polybutylene terephthalate (PBT) [7], polytrimethylene terephthalate (PTT) [7] and polyethylene furanoate (PEF) [8]. These engineering polymers can be produced either from non-renewable or renewable resources, the latter option being especially attractive as it offers the possibility of producing fully renewable composites with excellent mechanical and thermal properties [9,10]. However, the production of RF reinforced engineering polymer composites is especially challenging, and special care must be taken to limit the fibre degradation during processing to achieve good mechanical performance [10]. There are two main types of LCF and CF; natural fibres (NF), which are produced naturally by plants, or man-made cellulose fibres (MMCF), which are artificially produced from cellulosic materials. NF such as hemp, flax, ramie, jute and kenaf fibres are composed of cellulose, hemicellulose, lignin, pectin, waxes and other components (terpenes, oils, proteins, etc.), with the exact composition depending on the fibre type as well as other factors, such as growing conditions [4,11,12,13]. It is known that cellulose has the highest initial decomposition temperature (IDT) among these components [14]. On the other hand, MMCF are composed of pure cellulose. There are significant differences between the molecular structure and morphology of cellulose found in different NF and MMFC. The cellulose exhibits polymorphism and can be found in various crystalline structures, namely, cellulose I_α_, I_β_, II, III_I_, III_II_, IV_I_ and IV_II_. NF contain cellulose I_α_ and I_β_ polymorphs, while MMCF contains the thermodynamically more stable cellulose II polymorph [11]. Moreover, there are significant differences in the degree of polymerisation (*D_p_*) and the degree of crystallinity of cellulose found in various fibres. All these factors affect the decomposition behaviour of fibres, such as IDT, decomposition temperature (*T_d_*) and rate of decomposition (*R_d_*) [11,15]. In the past, some studies investigated the effect of the IM temperature on the properties of polymer composites reinforced with NF or MMCF. Feldmann investigated the effects of the IM temperature on the properties of PP reinforced with Cordenka MMCF. It was found that the mechanical properties decreased significantly at processing temperatures above 256 °C as a result of decreased fibre length due to mechanical stresses combined with lower fibre strength induced by the degradation. The author concluded that such composites can be processed at temperatures up to 250 °C without significant deterioration in mechanical properties [16]. Forsgren et al. investigated the effects of the IM temperature (170 °C, 200 °C and 230 °C) on the properties of ethylene-acrylic acid (EEA) copolymer reinforced with CF extracted from wood. Authors found that an increased IM temperature resulted in significant colour changes of the composites, but had no significant effect on the mechanical properties [17]. While these important works provided valuable insights into the influence of high processing temperatures on the reinforcing effect of RF, the studies were limited to only a single type of fibres. Therefore, the differences between various RF in terms of retention of reinforcing effect in composites processes at elevated temperatures are still unclear. Moreover, there are no established methods for the prediction of the reinforcing effect of RF based on fibre decomposition behaviour, which makes further development of RF reinforced engineering polymers very laborious.

The present study aimed to evaluate how increased processing temperatures influence the mechanical properties of thermoplastic composites reinforced with RF that have different thermal decomposition behaviour, and to establish relationships between the fibre decomposition behaviour and resulting mechanical properties of the composites. It was hypothesised that there are significant differences between various NF and MMCF in terms of retention of mechanical properties at elevated temperatures and that TGA can be a valuable tool for predicting this behaviour. PP was chosen as the matrix material because it has a wide processing window, allowing studies of fibre decomposition behaviour over a wide temperature range. Three different RF that are commonly used in thermoplastic composites were chosen as the reinforcements: two NF with different thermal decomposition behaviour (hemp and flax) and one MMCF (Lyocell). The results of the study should provide guidelines for the selection of the appropriate RF for the development of composites based on engineering thermoplastic polymers.

## 2. Materials

The injection moulding grade PP copolymer C765-15NA (Braskem, São Paulo, Brasil) was used as a polymer matrix. The PP had an MFR value of 15 g/10 min (230 °C, 2.16 kg) and a density of 0.900 g/cm^3^. PP copolymer grafted with maleic anhydride (PP-g-MA) Fusabond® P353 (Dupont, Wilmington, DE, USA) was used as a coupling agent. PP-g-MA had an MFR value of 22 g/10 min (160 °C, 0.325 kg) and a density of 0.904 g/cm^3^. The Lyocell fibres Tencel® (Lenzing, Lenzing, Austria) were kindly provided by Lenzing. The Lyocell fibres had a linear density of 1.7 dTex and a cutting length of 6 mm. The hemp and flax fibres were kindly provided by Ko-Si (Slovenj Gradec, Slovenia). The hemp and flax fibres were supplied in uncut form.

### 2.1. Material Preparation

#### 2.1.1. Milling

To have fibres in suitable length for pelletising the hemp and flax fibres were milled using a C 13.20s (Wanner Technik, Wertheim, Germany) granulator for injection moulding. The screen size was 5 mm.

#### 2.1.2. Pelletising

Both NF and MMCF have a very low bulk density, resulting in a challenging dosing behaviour using conventional processing equipment. Fibre pelletising techniques were developed to overcome these challenges by compressing the fibres into pellets that exhibit significantly higher bulk density [5,18]. The fibres were pelletised on a PTA 50 pelletiser (Tecno Aspira, Novedrate, Italy). The pelletiser had a 4 kW electric motor and a 6 mm die plate. Prior to pelletising, the fibres were conditioned to suitable moisture (15 wt.% for NF and 60 wt.% for MMCF). 

#### 2.1.3. Compounding

Composites were prepared using an LTE 20–44 (Labtech Engineering, Samut Prakan, Thailand) co-rotating twin-screw extruder. The extruder had a screw diameter of 20 mm and an L:D ratio of 44:1. The fibres in the form of pellets were dried prior to processing in a laboratory oven 100–800 (Memmert, Büchenbach, Germany) at 105 °C, to a moisture content of below 0.2 wt.%. The screw speed was 200 min^−1^. The extrusion temperature profile is summarised in Table 1.

#### 2.1.4. Injection Moulding

Test specimens according to ISO 527-2 1BA and ISO 178/179 were prepared using a CX 50–180 (Krauss-Maffei, München, Germany) injection moulding machine. The injection moulding machine had a screw diameter of 30 mm, an L:D ratio of 23.3:1 and a maximum clamping force of 500 kN. All materials were dried prior to processing in a 100–800 (Memmert, Büchenbach, Germany) laboratory oven at 105 °C, to a moisture content of below 0.2 wt.%. The samples were processed at temperatures of 180 °C, 200 °C, 220 °C, 240 °C and 260 °C. The exact temperature profiles for each temperature setting are summarised in Table 2. Other processing parameters are summarised in Table 3. All specimens were injection moulded using the same processing parameters, except for the switch-over point, which had to be adapted with increasing temperature to prevent under- or over-filling of the mould. Injection moulded composites were designated with abbreviations (e.g., PP-H-180), where PP corresponds to the matrix, H to the reinforcing fibres (hemp (H), flax (F) and Lyocell (L)), and 180 to the injection moulding temperature setting. 

### 2.2. Characterisation

#### 2.2.1. Simultaneous TGA/DSC

The decomposition behaviour of fibres and injection moulded specimens (ISO 527-2 1BA) was studied using a TGA/DSC 3+ (Mettler Toledo, Greifensee, Switzerland) simultaneous TGA/DSC apparatus in 40 µL aluminium crucibles. Non-isothermal measurements were performed by heating the samples from 40 °C to 550 °C, with a heating rate of 10 K/min in a nitrogen (N_2_) atmosphere (20 mL/min), followed by a 30 min isothermal segment in oxygen (O_2_) atmosphere (20 mL/min). Isothermal measurements were performed by heating the samples from 40 °C to the defined temperature, with a heating rate of 50 K/min in N_2_ atmosphere (20 mL/min) followed by a 20 min isothermal segment in the N_2_ atmosphere (20 mL/min).

#### 2.2.2. Optical Microscopy

Fracture surfaces of Charpy specimens were analysed using a VHX-6000 (Keyence, Osaka, Japan) digital microscope at 20× magnification.

#### 2.2.3. Colorimetry

The colour of the composites was determined on injection moulded specimens (ISO 178/179) using a CR-10 (Konica Minolta, Tokyo, Japan) colorimeter. Measurements were performed based on an International Commission on Illumination (CIE) L*a*b* colour space using a D65 standard illuminate and the observer angle of 10°.

#### 2.2.4. Tensile Tests

Tensile properties were determined using an Ag-X plus 10 kN (Shimadzu, Kyoto, Japan) universal testing machine according to ISO 527-1 standard. The gauge length was 50 mm, preload was 3 N and testing speed was 1 mm/min until 0.25% strain and 50 mm/min until breaking. 

#### 2.2.5. Charpy Impact Tests

The Charpy impact strength was determined using LY-XJJD5 (LIYI, Dongguan, China) pendulum impact tester, according to ISO 179-1 standard. Samples were tested using a 2 J pendulum.

#### 2.2.6. Dynamic Mechanical Analysis

Dynamic mechanical properties were determined using a DMA 8000 (Perkin Elmer, Waltham, MA, USA) dynamic mechanical analyser according to ASTM D5418 standard. Samples were tested in flexure using dual cantilever beam supports. The frequency was 1 Hz and the amplitude was 0.02 mm. Samples were heated from 30 °C to 160 °C, with a heating rate of 2 K/min. 

#### 2.2.7. Melt Flow Rate

The melt flow rate was determined using an LY-RR (LIYI, Dongguan, China) melt flow rate analyser according to ISO 1133-1 standard. The temperature was 230 °C and the load was 2.16 kg. 

## 3. Results

### 3.1. Simultaneous TGA/DSC

#### 3.1.1. Non-Isothermal Measurements of Fibres

**Figure 1:**
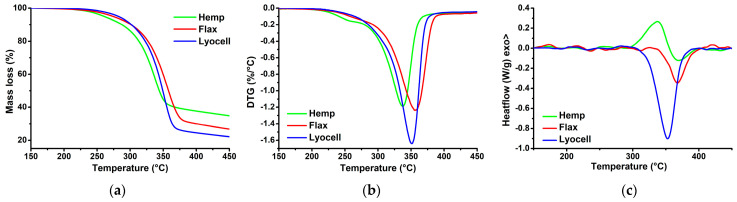
Non-isothermal TGA/DSC thermograms of hemp, flax and Lyocell fibres in the main decomposition region: (**a**) TGA thermograms; (**b**) DTG thermograms; (**c**) DSC thermograms.

The fibres were first measured using non-isothermal simultaneous TGA/DSC analyses to determine the general decomposition behaviour. Table 4 presents the thermal properties of fibres determined using TGA/DSC. All fibres exhibited three main mass loss steps, with the first mass loss region (40–150 °C) corresponding to moisture evaporation, the second mass loss region (150–550 °C) corresponding to the decomposition of cellulose (for NF also to pectin, hemicellulose, lignin and other impurities) and the third mass loss that occurred at 550 °C along with the shift from N_2_ to O_2_ atmosphere, was attributed to the decomposition of char formed during the main decomposition stage. Both hemp and Lyocell fibres had a moisture content (*w_m_*) of around 6%, while the flax fibres had a slightly lower *w_m_* of 4%. The higher *w_m_* of hemp and Lyocell fibres was ascribed to the higher content of pectin/hemicellulose of hemp fibres and a lower degree of crystallinity (*X_c_*) of both fibres compared to the flax fibres. Figure 1a presents TGA and Figure 1b presents derivative thermogravimetry (DTG) thermograms of fibres in the main decomposition region. The Lyocell fibre exhibited the highest initial decomposition temperature (IDT) (onset of TGA curve) of 320 °C, followed by the flax (−2.5 °C) and hemp fibre (−17.2 °C). Both hemp and flax fibre exhibited a slight shoulder in the DTG curve in the region of 210–280 °C that was ascribed to the decomposition of pectin/hemicellulose, which decomposes at lower temperatures than cellulose. The hemp fibres exhibited a much more prominent shoulder, indicating higher content of pectin/hemicellulose compared to flax fibres, which corresponds well with the higher moisture content of hemp fibres and findings reported in the literature [19]. These results are reflected in the temperature at 5% decomposition (*T*_5%_), where similar trends were found as for IDT, but with greater differences between the NF and MMCF. The early decomposition phase of NF and MMCF is influenced by different factors. NF besides cellulose contains various impurities that decompose at lower temperatures and can also, potentially, initiate the decomposition of the cellulose [15]. In addition, the supermolecular structure and morphology of the fibres also play an important role in the early decomposition stage. It is generally believed that the decomposition of cellulose occurs first in the amorphous regions [15,20,21,22]. Therefore, the early decomposition phase should also be closely correlated to the *X_c_* of the fibres. There are many different characterisation techniques and methods for estimating the *X_c_* of cellulose, and the results obtained can vary considerably [23]. However, values obtained using wide-angle X-ray scattering (WAXS) according to Segal [24] are usually in the range of 50%–70% for the hemp fibres [19,23], 60%–85% for the flax fibres [19,23] and 50%–70% [25,26] for the Lyocell fibres, with studies consistently reporting lower *X_c_* of hemp compared to flax [19,23]. The later stage of the decomposition was characterised by the decomposition temperature (*T_d_*), decomposition rate (*R_d_*) and decomposition enthalpy change (Δ*H_d_*) which correspond to the DTG peak temperature, DTG peak height and area under the DSC curve, respectively. Flax fibres exhibited the highest *T_d_* (356.6 °C), closely followed by Lyocell fibres (−4.0 °C), while hemp fibres had a much lower *T_d_* (−19.1 °C). Both hemp and flax fibres decomposed at a similar rate of around 0.12 min^−1^, while Lyocell fibres decomposed significantly faster (0.16 min^−1^). Since the initial stages of cellulose decomposition occur mainly in the amorphous region, the later stage is more correlated to the crystalline structure. In this respect, there are distinct differences between the NF and MMCF. Cellulose can exist in different polymorphic states, and while the cellulose found in NF has cellulose I structure, MMCF has a cellulose II structure. Moreover, the decomposition behaviour of cellulosic fibres is also related to the degree of polymerisation (DP) [15]. While estimated values of DP of hemp and flax fibres are in the range of 2500–5500 [27,28], and 2500–9500 [29,30,31], respectively, Lyocell has a DP of about 450 [32]. It has been shown that *R_d_* is correlated to the DP, which explains higher *R_d_* values of Lyocell fibres compared to NF [15]. Significant differences between the fibres were also observed in terms of Δ*H_d_*. Upon decomposition, hemp fibres exhibited an exothermic peak (Δ*H*_*d*1_) at 333.9 °C with an enthalpy change of 68.5 J/g and an endothermic peak (Δ*H*_*d*2_) at 367.9 °C with an enthalpy change of 15.4 J/g. On the other hand, flax and Lyocell fibres exhibited only endothermic peaks at 366.4 °C and 354.1 °C, with an enthalpy change of 47 J/g and 151.8 J/g, respectively. While cellulose decomposition is in general endothermic under inert atmosphere, it has been reported that in the case of hemp fibres the presence of lignin, which decomposes in a wide temperature range contributes to the exothermic decomposition of the cellulose [33]. NF exhibited considerably higher char content (*w_c_*) and ash content (*w_a_*) than Lyocell fibres, due to presence of lignin and inorganic components, respectively. 

#### 3.1.2. Isothermal Measurements of Fibres

**Figure 2:**
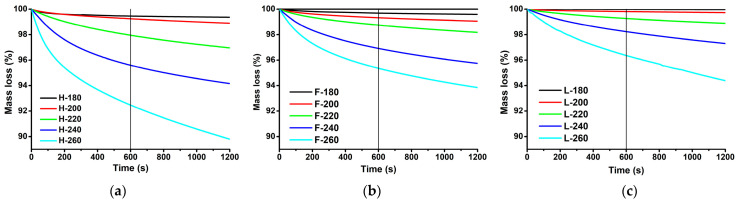
Mass loss of fibres as a function of time during the isothermal TGA measurements at different temperatures: (**a**) hemp fibres; (**b**) flax fibres; (**c**) Lyocell fibres.

The fibres were additionally characterised by isothermal TGA measurements at processing temperatures to evaluate the degree of decomposition during processing. The most important time window for investigation is the time around 10 min (600 s) as it is the estimated residence time of fibres during the IM. Figure 2 presents the mass loss of fibres as a function of time during the isothermal TGA measurements at different temperatures. The investigated fibres exhibited a non-linear mass loss, with faster decomposition rates at the beginning of the measurements and then more or less constant rates after a certain period. At the time of 10 min, hemp fibres exhibited the largest mass loss at the investigated temperatures, followed by flax and Lyocell fibres. This behaviour correlates well with the IDT and *T*_5%_ determined using non-isothermal TGA measurements. Although flax fibres had higher *T_d_* and lower *R_d_* than Lyocell fibres, they still exhibited a higher mass loss over the whole examined exposure time even at a temperature of 260 °C. However, it was observed that higher mass losses of flax fibres at this temperature originate from the early phase of the measurement, whereas in the later phase the Lyocell exhibited a faster rate of mass loss, evident by a larger slope of the curve. This effect may be ascribed to the pectin/hemicellulose in the flax fibre, which decomposes at lower temperatures, resulting in a significant mass loss of the fibre in the early stage, while higher DP and *X_c_* of flax reflects in a lower decomposition rate in the later stage of the measurement. It was found that the temperature of exposure had a much higher effect on the fibre mass loss compared to the exposure time at least for practical implications considering melt blending of RF with polymers. 

### 3.2. Optical Microscopy

**Figure 3:**
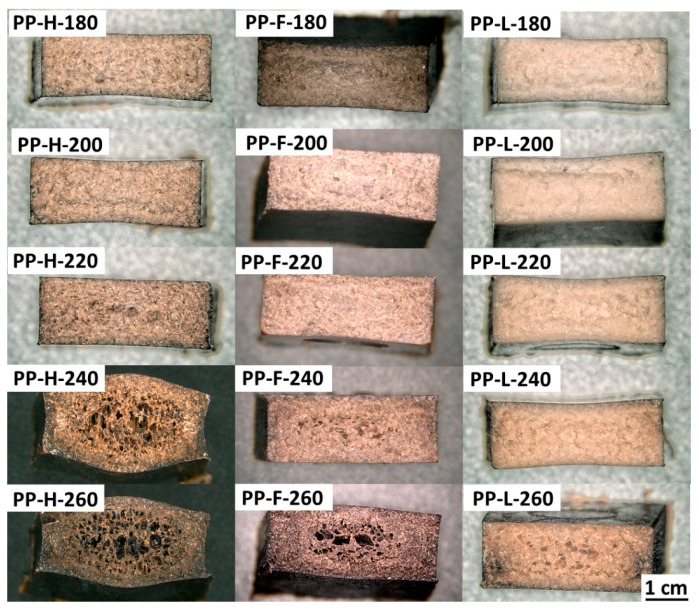
Micrographs of the fracture surfaces of composites processed at different temperatures.

The fracture surfaces of the Charpy specimens were examined using OM to gain better insight into the structure-property relationships of the composites processed at different temperatures (micrographs of the surfaces are presented in Figure 3). The micrographs revealed that the investigated samples processed at lower temperatures exhibited homogeneous and compact structures, combined with a concave cross-section shape, which was due to the shrinkage of the polymer matrix during the cooling phase of injection moulding. Above a certain processing temperature, which depended on the reinforcing fibre, the samples started to exhibit a porous structure and a rectangular or even convex cross-section shape, both phenomena being attributed to the formation of decomposition gasses during processing. In this respect, significant differences were found between the composites reinforced with different fibres. The samples of the composites reinforced with hemp fibres processed at 220 °C were slightly porous and had a rectangular shape, while the samples processed at 240 °C and 260 °C had high porosity and a convex cross-sectional shape. The samples of the flax fibre reinforced composites began to exhibit porosity at processing temperatures above 240 °C, where similar structure and cross-sectional shape was observed to the hemp fibre reinforced composites processed at 220 °C, while the flax fibre reinforced composites processed at 260 °C exhibited less porosity than the hemp fibre reinforced composites processed at 240 °C. On the other hand, Lyocell fibre reinforced composites were only slightly porous at a processing temperature of 260 °C and no significant porosity was observed in the samples processed at lower temperatures. 

### 3.3. Colorimetry

**Figure 4:**
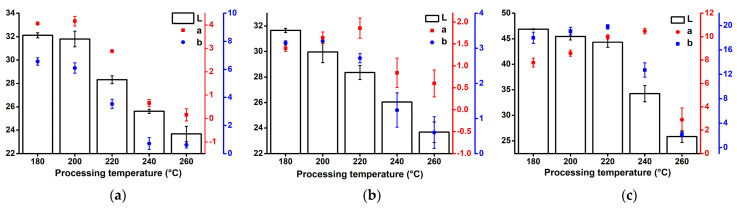
Colorimetry results (L*a*b*) of the composites processed at different temperatures: (**a**) PP/hemp; (**b**) PP/flax; (**c**) PP/Lyocell.

Colorimetry has been shown to be a valuable tool for monitoring the thermal degradation of CF reinforced composites. It has been shown that with increasing IM temperature, L* and b* values of CF composites decrease [34]. In this study, the L*a*b* colour space was used to determine the effect of the processing temperature on the composite colour. The L* value represents the perceptual lightness (0—black, 100—white), the a* value is related to the green-red opponent colours (negative values towards green, positive values towards red) and the b* value is related to the blue-yellow opponents (negative values towards blue, positive values towards yellow). Figure 4 presents the results of colorimetry of composites processed at different temperatures. As the processing temperature increased, the composites became darker in colour. Hemp fibre composites started to exhibit darker colour at a processing temperature of 220 °C, at which a significant decrease in the L* value was observed that persisted with increasing temperature. For the flax fibre composites, the significant decrease in the L* value started at a processing temperature of 200 °C, which persisted more or less constantly as the processing temperature increased. Lyocell fibre composites were overall much lighter in colour (higher L* values) than NF composites. Furthermore, Lyocell composites exhibited only a slight decrease in L* up to a processing temperature of 240 °C, at which a substantial decrease of L* was observed. Important differences between the composites were also observed in terms of colouration and discolouration (changes in a* and b* values). Hemp composites exhibited discolouration characterised by the decrease in a* and b* values with increasing processing temperatures, except for the a* value at a temperature of 200 °C, which was approximately at the same level as for composites processed at 180 °C. On the other hand, both flax and Lyocell composites exhibited slight colouration with increasing temperature up to a certain point, then discolouration was observed. 

### 3.4. Tensile Tests

**Figure 5:**
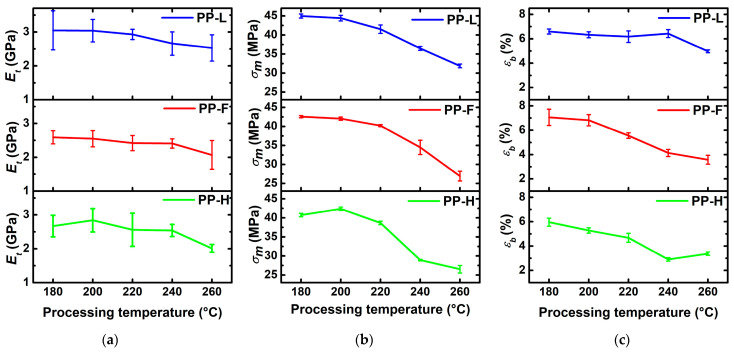
Tensile properties of composites processed at different temperatures: (**a**) tensile modulus; (**b**) tensile strength; (**c**) strain at break.

The mechanical properties of NF or MMCF composites are related to the properties of the matrix and fibres, the fibre volume fraction, the interactions between the components and various microstructural features (distribution, dispersion and orientation of fibres and porosity) [1]. The tensile modulus of the composite is mainly related to the modulus and volume fractions of the matrix and fibres, fibre aspect ratio and microstructure of the composites [35]. The tensile strength of the composites also depends on the same factors (if substituting the modulus of components with strengths) and additionally on the interfacial adhesion between the matrix and the fibres. However, the tensile strength of the fibres plays only plays an important role if the fibres are perfectly aligned in the loading direction, fully surrounded by the matrix, and longer than the critical fibre length (*l_c_*). Otherwise, failure occurs due to fibre pullout instead of fibre breakage [36]. Figure 5 presents tensile modulus (*E_t_*), tensile strength (*σ_m_*) and strain at break (*ε_b_*) of the composites processed at different temperatures. The tensile properties of neat PP processed at 180 °C were *E_t_* = 1.29 GPa, *σ_m_* = 25.8 MPa and *ε_b_* = 39.6%. The introduction of fibres into PP increased *E_t_* and *σ_m_* to values in the range of 2.6 GPa–3.0 GPa and 41 MPa–45 MPa, respectively, and decreased *ε_b_* to the values in the range of 6.0%–7.1% due to the fibre reinforcing effect. The *E_t_* of NF reinforced composites was approximately in the same range, while Lyocell fibre composites exhibited a slightly higher *E_t_.* Significant differences were observed in terms of *σ_m_*, with Lyocell fibres exhibiting the strongest reinforcing effect, followed by flax and hemp fibres. These differences can be explained by different mechanical, geometrical and surface properties of the fibres, which either directly affect the mechanical properties of the composite or indirectly by influencing the resulting microstructure of the composite and the morphology of the matrix. When investigating the effect of the processing temperature on the tensile properties of the composites, it can be observed that the processing temperature generally had the most significant effect on the *σ_m_*, which decreased with increasing temperature. With increasing temperature, the composites exhibited a slight decrease in *σ_m_* up to 220 °C, except for the hemp fibre composites, where *σ_m_* increased at 200 °C. At the processing temperature of 240 °C, a significant decrease in *σ_m_* was observed, the extent of which depended on the fibre type. When comparing *σ_m_* of composites processed at 180 °C and 240 °C, the most significant decrease was observed in the hemp fibre composites (−29.0%), followed by flax (−19.0%) and Lyocell (−18.9%) fibre composites. At 260 °C, *σ_m_* was further reduced to 26.5 MPa (−34.9%), 26.9 MPa (−36.9%) and 31.9 MPa (−29.1%) for hemp, flax and Lyocell fibres, respectively. At this processing temperature, the NF composites exhibited *σ_m_* comparable to the neat matrix, while Lyocell fibres still had a reinforcing effect. Decreasing trends were much less evident with respect to *E_t_*. First, the measurement error was much higher, and second, although both *σ_m_* and *E_t_* depend on the aspect ratio of the fibres, the effect on the *E_t_* is much smaller, as stress can be efficiently transferred to the shorter fibres at lower stress levels. As the processing temperature increases, several factors can contribute to the tensile properties of the composite. On the one hand, increased temperature leads to a decrease in viscosity, which in turn affects fibre distribution and dispersion behaviour, as well decreases shear stresses on the fibres, thus preventing fibre shortening. On the other hand, a higher processing temperature leads to degradation of the fibres which in turn leads to reduced fibre strength and formation of gaseous products. Reduced fibre strength can have a direct effect on the *σ_m_* value of the composite or indirectly as a result of more severe fibre damage.

### 3.5. Charpy Impact Test

**Figure 6:**
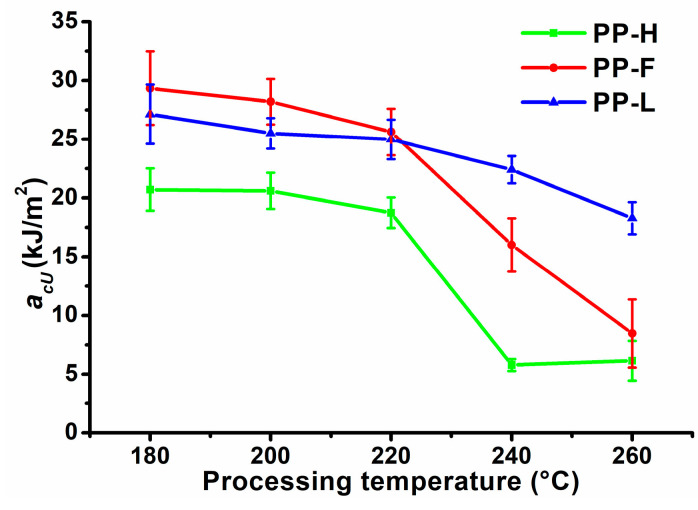
Unnotched Charpy impact strength of composites processed at different temperatures.

The unnotched impact strength of fibre reinforced thermoplastic composites is related to the same factors as *σ_m_*, although to different degrees. However, while the introduction of short fibres into the thermoplastic matrix leads to an increase in *σ_m_*, it generally decreases unnotched impact strength as interfacial cracks develop at the fibre ends, propagating first along the matrix-fibre interface and eventually through the matrix, leading to composite failure [37,38]. Figure 6 presents Charpy unnotched impact strength (*a_cU_*) of composites processed at different temperatures. The neat PP matrix did not break during the impact test, while at a processing temperature of 180 °C flax fibre composites exhibited the highest *a_cU_* (29.4 kJ/m^2^) followed by Lyocell (27.1 kJ/m^2^) and hemp fibre composites (20.7 kJ/m^2^). With increasing processing temperature, *a_cU_* followed a similar trend as to *σ_m_*, which is not surprising since both properties depend on the same factors. Up to a processing temperature of 220 °C, the composites exhibited only a slight decreasing trend of *a_cU_*. Similar to *σ_m_*, a significant decrease of *a_cU_* was observed at a processing temperature of 240 °C, which depended on the fibre type. At 240 °C, the composites reinforced with hemp fibres exhibited the largest decrease of *a_cU_* (−72%), followed by composites reinforced with flax (−45%) and Lyocell fibres (−17%). An increase of the processing temperature to 260 °C resulted in an additional decrease of *a_cU_* for flax and Lyocell fibre reinforced composites, while *a_cU_* did not decrease further for hemp fibre composites. The decrease of *a_cU_* at increased processing temperatures was attributed to several factors: increased porosity due to gas formation during fibre decomposition, decreased crack initiation energies due to more fibre ends and decreased crack propagation energies due to weaker and shorter fibres.

### 3.6. Dynamic Mechanical Analysis

**Figure 7:**
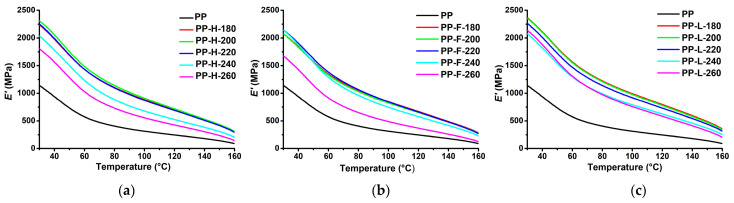
Storage modulus as a function of the temperature of composites processed at different temperatures: (**a**) PP/hemp; (**b**) PP/flax; (**c**) PP/Lyocell.

The dynamic mechanical analysis provides data on the viscoelastic behaviour of polymers. The storage modulus (*E*′) is related to the storage of energy and represents the elastic component, the loss modulus (*E*″) is related to the dissipated energy and represents the viscous component, while the damping factor (tan *δ*) is a ratio of *E*″ to *E*′ and is a measure of the damping performance of the material. Figure 7 presents *E*′ as a function of the temperature of PP and composites processed at different temperatures. The addition of fibres significantly increased *E*′ over the entire investigated temperature range, which can be attributed to the reinforcing effect of the fibres. At 30 °C, composites processed at low-temperatures exhibited an *E*′ approximately twofold higher compared to neat matrix, while at higher measurement temperatures the relative increase in *E*′ was greater because the stiffness of fibres is less affected by temperature compared to the matrix, and the molecular motion of the highly mobile PP chains is restricted by the stiff fibres. At 30 °C, Lyocell fibre composites processed at low temperatures had the highest *E*′ value, followed by hemp and flax fibre composites, respectively, which unsurprisingly correlates well with the *E_t_* results since both quantities represent the stiffness of the sample. The hemp fibre reinforced composites exhibited a decrease in *E*′ at a processing temperature of 240 °C and a further decrease at 260 °C. In contrast, for the flax fibre composite, *E*′ decreased significantly at 260 °C, while the composite processed at 240 °C had slightly lower *E*′ than the composites processed at low temperatures only above about 50 °C. Lyocell fibre composites exhibited a decrease in *E*′ with increasing processing temperature, however, the decrease was only minor up to the processing temperature of 240 °C. Similar to *E_t_*, the *E*′ of the composites at room temperature is much less sensitive to the effects of fibre degradation occurring at higher processing temperatures compared to *σ_m_* and *a_cU_*. However, at higher measurement temperatures, the effect of fibre degradation becomes much more pronounced. While a shorter fibre length has little effect on *E*′ at lower temperatures, where polymer chain mobility is rather low, it has a significant effect on *E*′ at temperatures closer to the melting point of the matrix as longer fibres are more effective in restricting the molecular motion of the highly mobile PP chains. 

**Figure 8:**
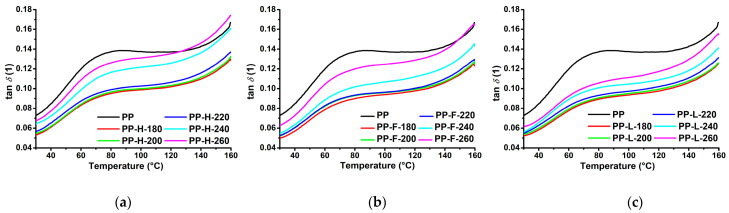
Damping factor as a function of the temperature of composites processed at different temperatures: (**a**) PP/hemp; (**b**) PP/flax; (**c**) PP/Lyocell.

Figure 8 presents tan *δ* as a function of the temperature of neat PP and of composites processed at different temperatures. All samples exhibited a slight tan *δ* peak at a temperature around 70 °C, corresponding to the α-transition of the PP matrix associated with the relaxation of the confined amorphous PP chains into the crystalline phase [39]. The incorporation of fibres into a thermoplastic matrix usually increases *E*′ due to reinforcing effects of the fibres, and increases *E*″ due to heat dissipation effects at the matrix/fibre and fibre/fibre interfaces. However, *E*′ usually increases more than *E*″, resulting in a decreased damping performance, which is reflected in lower values of tan *δ*. In our case, composites processed at 180 °C exhibited significantly decreased tan *δ* over the whole examined temperature range. As the processing temperature increased up to 220 °C, tan *δ* increased only slightly. At a processing temperature of 240 °C, the composites exhibited a significant increase in tan *δ*, with a further increase observed at 260 °C, both of which were especially evident above the onset of the α-transition with its magnitude dependent on the fibre type. Similar trends were observed for the damping properties as for the other mechanical properties. Significant changes were observed at processing temperatures above 220 °C, with composites reinforced with hemp fibres exhibiting the greatest changes, followed by flax and Lyocell fibres. Composites reinforced with hemp and flax fibres exhibited better damping performance than a neat PP matrix at temperatures above 130 °C and 150 °C, respectively, despite having a higher *E*′ in this temperature range. The reason for this is that severely damaged fibres lead to only a small increase of *E*′, especially at high temperatures, while the presence of matrix/fibre and fibre/fibre interfaces and the associated heat dissipation effects still lead to high *E*″ values. 

### 3.7. Melt Flow Rate

**Figure 9:**
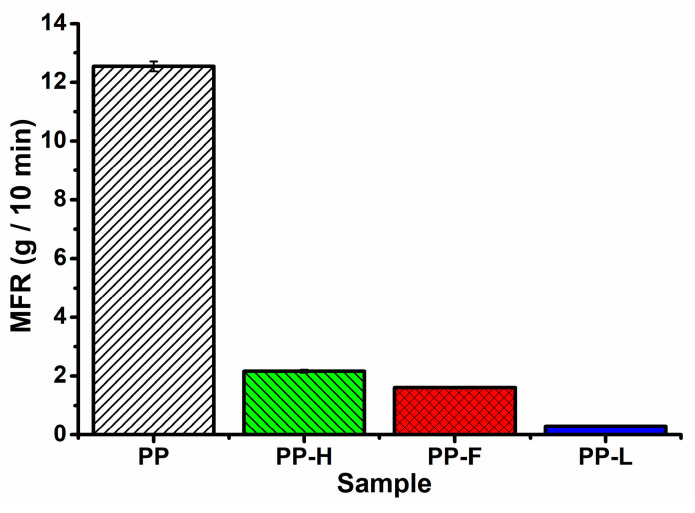
Melt flow rate of neat PP and composites reinforced with hemp, flax and Lyocell fibres.

The flow behaviour of neat PP and composites was evaluated using a melt flow rate that corresponds to the viscosity at a low shear rate. MFR results are presented in Figure 9. The addition of fibres significantly decreased the MFR of neat PP in the order of Lyocell < flax < hemp fibres. The differences in the MFR values of the composites were attributed to the different aspect ratios of the fibres in the composites. It is well known that the aspect ratio of the fibres has a strong influence on the viscosity of the composites, especially at low shear rates, as fibres with higher aspect ratio result in worse fibre dispersion, while at the same time they are more difficult to orient in the flow direction [40]. The decrease in MFR corresponds well with the *σ_m_* values of composites processed at low temperatures, which also dependents on the fibre aspect ratio.

### 3.8. Relationships between Thermal Properties of Fibres and Mechanical Properties of Composites

**Figure 10:**
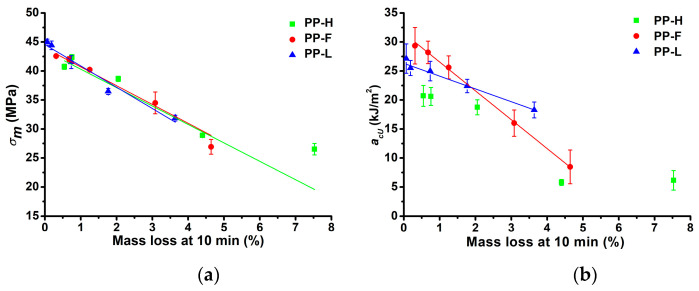
Correlations between the mass loss of fibres at 10 min of isothermal TGA measurements and mechanical properties of composites: (**a**) tensile strength; (**b**) Charpy impact strength.

Correlations between the mechanical and thermal properties of composites processed at different temperatures and fibre mass loss at 10 min (Δ*m*_10_) of isothermal TGA measurements at the corresponding temperatures were modelled using linear regression. Δ*m*_10_ was chosen because it corresponds to the approximate residence time of the composites in the injection moulding process. Significant correlations were found for *σ_m_* and *a_cU_*. Figure 10a presents *σ_m_* of the composites as a function of fibre Δ*m*_10_. It was found that the tensile strength of composites exhibited an inverse linear relationship to the fibre Δ*m*_10_ in the investigated temperature range. Hemp fibre reinforced composites exhibited the lowest correlations between *σ_m_* and Δ*m*_10_ with an adjusted coefficient of determination (*R*^2^*_adj_*) of 91.6%. Composites reinforced with flax and Lyocell fibres exhibited higher correlations between *σ_m_* and Δ*m*_10_, with *R*^2^*_adj_* values of 95.3% and 95.6%, respectively. Figure 10b presents *a_cU_* of the composites as a function of Δ*m*_10_. The *a_cU_* value of flax and Lyocell composites were almost perfectly correlated to the Δ*m*_10_ value in an inverse linear relationship, especially when considering the standard deviations of measurements, with *R*^2^*_adj_* values of 99.8% and 98.2%, respectively. On the other hand, the hemp fibre composites exhibited a non-linear relationship between *a_cU_* and Δ*m*_10_. It seems that the linear relationship between the mechanical properties (*σ_m_* and *a_cU_*) of the composites and Δ*m*_10_ is valid, but only up to a certain level of fibre degradation, above which the fibres are degraded to an extent that they no longer provide a reinforcing effect.

## 4. Conclusions

Composites based on PP, reinforced with hemp, flax and Lyocell fibres were compounded, injection moulded at various temperatures and characterised. The thermal decomposition behaviour of fibres was evaluated and correlated with the mechanical properties of the composites. Non-isothermal TGA measurements revealed significant differences in the decomposition behaviour of the investigated fibres. NF started to decompose at lower temperatures in comparison to Lyocell fibres, while the latter exhibited a higher decomposition rate in the main decomposition region. Flax fibres exhibited better overall thermal stability than hemp fibres. These differences were also reflected in isothermal TGA measurements, where Lyocell fibres exhibited the lowest mass loss at the investigated temperatures, followed by flax and hemp fibres. Lower thermal stability of NF in the initial decomposition region was attributed to the presence of non-cellulosic compounds, which began to decompose at low temperatures, and thus further initiated the decomposition reaction of the fibres. Lyocell fibres offered the best mechanical performance, especially at higher processing temperatures, followed by flax and hemp fibres. The investigated fibres offered a good reinforcing effect up to a processing temperature of 220 °C, whereas a significant deterioration of the properties of the composites was observed at 240 °C. Increased processing temperatures had a strong effect on the tensile strength, strain at break, Charpy impact strength and stiffness of the composites at elevated temperatures, while the stiffness at about room temperature was only slightly affected. The decrease of mechanical properties was accompanied by increased darkness (decreased L* values) of the composites. High correlations were found between the mechanical properties (tensile strength and Charpy impact strength) of the composites processed at elevated temperatures and the decomposition behaviour of fibres determined using the isothermal TGA measurements. Both properties exhibited an inverse linear relationship to the fibre mass loss at 10 min. However, the relationship was only valid to the level of fibre degradation, at which fibres still offered a reinforcing effect. While similar previous works reported a decrease of mechanical properties of RF reinforced composites at temperatures above 256 °C or even no deterioration of mechanical properties with increased temperature, the present study found that the retention of mechanical properties of composites is highly dependent on the decomposition behaviour of RF. In this respect, pure CF with a high degree of crystallinity should be advantageous, since typical impurities present in LCF decompose at lower temperatures and can initiate the decomposition of cellulosic components, while the first stages of cellulose decomposition take place mainly in the amorphous regions. The results suggest that TGA is a valuable tool for evaluating RF and possibly their modifications, in terms of suitability for reinforcing engineering polymers. While this work provides first insights into the relationships between fibre decomposition behaviour and resulting properties of composites processed at elevated temperatures, further studies are needed to verify whether the conclusions generalise to other RF as well as to composites based on engineering polymers. 

## Figures and Tables

**Table 1:** Extrusion temperature profile for the preparation of composites.

Zone	Die	11	10	9	8	7	6	5	4	3	2	1
**Temperature (°C)**	190	195	200	200	190	190	190	180	180	170	160	150

**Table 2:** Exact temperature profiles used for injection moulding of test specimens.

Temperature Setting	Nozzle (°C)	T_4_ (°C)	T_3_ (°C)	T_2_ (°C)	T_1_ (°C)
180 °C	180	170	160	150	140
200 °C	200	190	180	170	160
220 °C	220	210	200	190	180
240 °C	240	230	220	210	200
260 °C	260	250	240	230	220

**Table 3:** Injection moulding parameters used for the preparation of the test specimens.

Parameter	Value
Injection velocity (mm/s)	50
Switch-over point (mm)	6–9
Metering stroke (mm)	17
Packing pressure (MPa)	40
Packing time (s)	8
Screw speed (min^−1^)	100
Backpressure (MPa)	10
Mould temperature (°C)	20
Rest cooling time (s)	5

**Table 4:** Thermal properties of hemp, flax and Lyocell fibres determined using TGA/DSC.

Fibre	*w_m_* (%)	IDT (°C)	*T*_5%_ (°C)	Δ*Y* (%)	*T_d_* (°C)	*R_d_* (min^−1^)	Δ*H*_*d*1_ (J/g)	Δ*H*_*d*2_ (J/g)	*w_c_* (%)	*w_a_* (%)
Hemp	6.0	302.8	260.3	69.2	337.5	0.12	68.5	−15.4	22.2	2.6
Flax	4.0	317.5	274.3	76.5	356.6	0.12	−47.0	/	18.2	2.3
Lyocell	5.9	320.0	283.2	80.4	351.6	0.16	−151.8	/	13.7	0.1

## Data Availability

Not Applicable.
